# Making patient blood management the new norm(al) as experienced by implementors in diverse countries

**DOI:** 10.1186/s12913-021-06484-3

**Published:** 2021-07-02

**Authors:** Axel Hofmann, Donat R. Spahn, Anke-Peggy Holtorf, PBM Implementation Group, James Isbister, Jeff Hamdorf, Linda Campbell, Bruno Benites, Gustavo Duarte, Guillermo Rabello, Hongwen Ji, Lihui Wei, Visnja Ivancan, Natasa Kovac, Tina Tomic Mahecic, Chara Matsouka, Bairaktari Aggeliki, Gafou Anthi, Alexandros Charalabopoulos, David Attalah, Samia Jebara, Rabih Chahine, Ángel Augusto Pérez Calatayud, Ángel Fernando Galvan Garcia, Miguel Ayala, Bettina Torres Pérez, Jong Hoon Park, Young Woo Kim, Jeong Jae Lee, Tae Hyun Um, Hind A.Al-Humaidan, Ammar Al Sughayir, Khalid Batarfi, Salwa Hindawi, Vernon Louw, Jackie Thompson, Neslihan Alkis, Serdar Gunaydin, Berrin Gunaydin

**Affiliations:** 1 Institute of Anesthesiology, University and University Hospital of Zurich, Zurich, Switzerland; 2 University of Western Australia Faculty of Health and Medical Sciences, Perth, Australia; 3 Health Outcomes Strategies GmbH, Colmarerstrasse 58, CH4055 Basel, Switzerland; 4 Faculty of the College of Pharmacy, University of Utah, Salt Lake City, UT USA

**Keywords:** Patient blood management, Transfusion, Patient outcomes, Practice change, Culture change, Implementation

## Abstract

**Background:**

Patient blood management (PBM) describes a set of evidence-based practices to optimize medical and surgical patient outcomes by clinically managing and preserving a patient’s own blood. This concepts aims to detect and treat anemia, minimize the risk for blood loss and the need for blood replacement for each patient through a coordinated multidisciplinary care process. In combination with blood loss, anemia is the main driver for transfusion and all three are independent risk factors for adverse outcomes including morbidity and mortality. Evidence demonstrates that PBM significantly improves outcomes and safety while reducing cost by macroeconomic magnitudes. Despite its huge potential to improve healthcare systems, PBM is not yet adopted broadly. The aim of this study is to analyze the collective experiences of a diverse group of PBM implementors across countries reflecting different healthcare contexts and to use these experiences to develop a guidance for initiating and orchestrating PBM implementation for stakeholders from diverse professional backgrounds.

**Methods:**

Semi-structured interviews were conducted with 1–4 PBM implementors from 12 countries in Asia, Latin America, Australia, Central and Eastern Europe, the Middle East, and Africa. Responses reflecting the drivers, barriers, measures, and stakeholders regarding the implementation of PBM were summarized per country and underwent qualitative content analysis. Clustering the resulting implementation measures by levels of intervention for PBM implementation informed a PBM implementation framework.

**Results:**

A set of PBM implementation measures were extracted from the interviews with the implementors. Most of these measures relate to one of six levels of implementation including government, healthcare providers, funding, research, training/education, and patients/public. Essential cross-level measures are multi-stakeholder communication and collaboration.

**Conclusion:**

The implementation matrix resulting from this research helps to decompose the complexity of PBM implementation into concrete measures on each implementation level. It provides guidance for diverse stakeholders to design, initiate and develop strategies and plans to make PBM a national standard of care, thus closing current practice gaps and matching this unmet public health need.

**Supplementary Information:**

The online version contains supplementary material available at 10.1186/s12913-021-06484-3.

## Background

Of the millions of patients hospitalized yearly, a large proportion is anemic at admission. Preoperative anemia rates range from 20 to 75% [1], and hospital acquired anemia often adds to the problem [2]. In most cases, anemia is not considered a clinically significant condition, remains unnoticed, and therefore uncorrected in hospitalized patients.

However, a large body of evidence shows that anemia, blood loss, and transfusion are independent risk factors for adverse outcomes including morbidity, mortality and average length of hospital stay [3–6]. Patient blood management is defined by the WHO as ”a set of evidence-based practices to optimize medical and surgical patient outcomes through preservation of the patient’s own blood” [7]. The International Foundation for Patient Blood Mangement specifies, that “Patient Blood Management (PBM) is an evidence-based bundle of care to optimize medical and surgical patient outcomes by clinically managing and preserving a patient’s own blood” [8]. Patient blood management rests on three pillars: diagnosis and treatment of anaemia (especially iron deficiency anaemia), minimization of blood loss, and avoidance of unnecessary transfusions. In addition to being a fundamental element of good clinical practice in transfusion, it plays a key role in primary health care. The multi-professional, multimodal, and individualized approach involves general practitioners, hematologists, anesthesiologists, intensive care specialists, surgeons, and others. The term ‘Patient Blood Management’ was coined in 2005 [9], but the concept has been emerging since a much longer time [10, 11]. Meanwhile, large multicentric observational studies and randomized controlled trials demonstrated that Patient Blood Management significantly improves morbidity, mortality, and average length of hospital stay, while reducing overall cost of care [12–15]. Clinical thought leaders urge that Patient Blood Management should be implemented as standard of care, and reduction of allogeneic blood product utilization should serve as a marker for success [16, 17]. In 2010, the World Health Organization (WHO) endorsed Patient Blood Management [18] and the fourth Strategic Objective of the ‘WHO Action framework for blood products 2020-2023’ released in February 2020 calls for ‘Effective implementation of patient blood management’ [7].

However, despite compelling evidence and ongoing WHO policy drive, practical guidance for healthcare providers and national authorities [16, 19–21] and clinical guidelines and recommendations across numerous specialties and national health systems [17, 22–30], implementation of Patient Blood Management is still far behind the expectations for good and safe clinical practices.

The implementation of Patient Blood Management is hampered by barriers mostly related to the difficulty of changing traditional “physicians’ attitudes” towards transfusion [31] and “transfusion behavior” [32–34]. Even hard-hitting crises such as the HIV-pandemic in the 1970s and 1980s with tens of thousands infected from contaminated donor blood, the huge death toll, billions of dollars in financial losses from lawsuits and compensations and criminal charges [35] only had a transient impact on changing long standing transfusion practice [36]. What was called at the time “transfusion alternative strategies” showed compelling results and could have been helpful to reduce overall blood utilization with similar outcomes [37–40], but went largely unnoticed [3]. Instead, the focus remained solely on improving blood product safety through introducing donor blood testing methods with unprecedented cost per quality adjusted life year (QALY) between 4.7 and 11.2 million US-$, representing 94–224 times the then commonly accepted threshold in public health decision making (50,000 US-$/QALY) [41, 42]. Meanwhile, and despite rapidly accumulating clinical evidence for adverse transfusion outcomes and favorable Patient Blood Management outcomes [43], numerous Patient Blood Management guidelines [17, 22–30], WHO endorsement [18], call for Patient Blood Management [7], and several national policy recommendations, the global implementation of Patient Blood Management is still alarmingly slow. Huge inter-center and inter-country transfusion variability indicates, that blood utilization is rather driven by culture and behavior than evidence [33, 34, 44–46].

Continuing to ignore the cumulative evidence puts life, well-being and safety of millions of hospitalized patients at risk. Delaying Patient Blood Management implementation also means that healthcare systems forego savings of macro-economic magnitudes from a system-wide implementation of Patient Blood Management [15]. This is even more alarming in countries striving towards Universal Healthcare Coverage and with severe resource constraints. In 2016, Eichbaum et al. compared the Patient Blood Management implementation status in four countries using a six-questions survey and observed considerable variation between countries driven both by differences in health contexts and disparities in resources [47]. They concluded that comparing Patient Blood Management strategies across low-, middle-, and high-income countries should foster mutual learning and implementing innovative, evidence-based strategies for improvement.

Following this recommendation, a more in-depth questionnaire was developed in this study to gather, through interviews, the experiences of a diverse group of implementors of Patient Blood Management across countries with different economic and healthcare contexts. The first aim was to describe the status-quo and chosen implementation approach in each of the surveyed countries, and to extract the drivers, barriers, measures, and stakeholders to be involved. The second aim of the study was to analyze this information and synthesize it into an implementation framework for Patient Blood Management which can serve as a comprehensive guidance how to implement Patient Blood Management.

## Methods

Semi-structured interviews mostly lasting 45–60 min were conducted between November 2019 and May 2020 with a multi-disciplinary group of 36 Patient Blood Management implementors leading the implementation of Patient Blood Management in their respective environment. Ten countries from Latin America, Central and Eastern Europe, Asia, Middle East and Africa were selected to reflect experiences from countries with different levels and types of healthcare resources and system (national/ private funders, public / private providers), and different developmental stages of Patient Blood Management (from early stage to more advanced). In addition, Australia was chosen as a reference country, where Patient Blood Management is adopted broadly and supported through public health authorities since 2008 and through National Patient Blood Management Guidelines since 2009 [15, 48, 49]. Likewise, a Swiss reference case was included, where Patient Blood Management is sustainably implemented across a leading hospital (University Hospital of Zürich). All interviewees were actively involved in implementing Patient Blood Management, and they were selected from the network of the authors, the International Foundation Patient Blood Management [50], and the local networks of the industry or other interviewees. The selection aimed to represent different clinical disciplines (e.g., hematologists, anesthesiologists, surgeons) and perspectives (e.g., clinical specialists, blood bank, policy, Patient Blood Management coordinator, industry). All interviews followed the structure of a newly developed questionnaire (Additional File 1). One question required rating of predefined barriers between 0 (not important) and 4 (very important). To allow the respondent to provide potentially unexpected answers, all other nine questions were formulated open without prompting specific answers. The survey was piloted with 11 interviewees and then fully rolled-out after minor improvements in language and sequence of questions (survey flow). Most interviews were conducted via web-communication (GoToMeeting™) by a single interviewer (AP Holtorf, Dr. rer. nat, female, without pre-existing relationship to the interviewees) in English language, two interviews were conducted by a second qualified male interviewer in Chinese language after detailed briefing by the main interviewer. The interview questionnaire was provided to the interviewees at least 1 week before the interviews. During the interviews, the interviewees verbally consented to note-taking, recording, and publication of the results. The notes were revised using the recordings and the interviewees had the opportunity to review, correct or complement their initial responses. The COREQ checklist was applied to document transparent reporting of this interview-based qualitative study and the completed form is available as Additional File 2 [51]. Qualitative content analysis was performed for analysis and synthesis following published guidance [52, 53]: 1.) Responses per country were extracted to a structured summary document (from two to four interviews per country except for Switzerland with one). 2.) Responses from all countries regarding status-quo, approach of the implementation, and 3.) drivers, barriers, measures, and stakeholders for Patient Blood Management were transferred in an electronic spreadsheet and coded guided by the items mentioned by the interviewees (grounded theory approach). 4.) The coded responses from step three were evaluated for the frequency of mentions (frequency analysis). 5.) Accelerating and inhibiting factors were pooled and translated into implementation measures (re-coding). 6.) Using an axial coding approach [54], the measures were classified by the interventional levels (policy/government, funding, research, healthcare provision, training/education, and public / patients). Steps 1 to 5 were conducted by the main interviewer and step 6 collaboratively by the authors.

## Results

### Demographics

Thirty-six Patient Blood Management implementors, named “Patient Blood Management Implementation Group” with 15 women and 21 men from 12 countries, were interviewed following 11 pilot interviews (total of 47). The respective perspectives are depicted in Table 1.

**Table 1:**
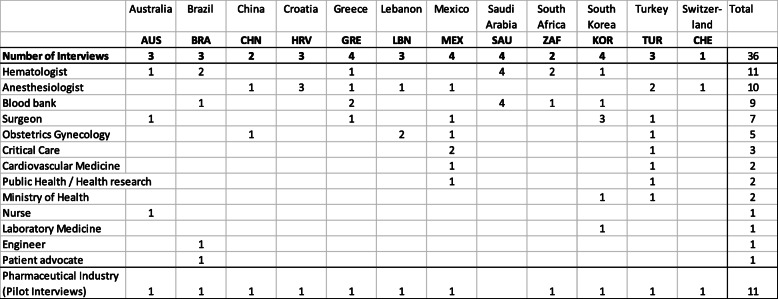
Demographics of the interviewees. (1 expert could represent more than 1 perspectives. Therefore, the numbers in the columns can add up to more than the number of experts). The survey was piloted with representatives of pharmaceutical companies being actively involved in Patient Blood Management (Abdi Ibrahim, Genesis Pharma, Hikma, Sandoz Pharma, Takeda, Vifor Pharma)

### Current status and approach in implementing patient blood management (question 3)

The country-level responses for the current level of Patient Blood Management implementation and the approaches (top-down, bottom-up, or both approaches simultaneously) are summarized in Table 2*.* Australia, after initial bottom-up implementation in several leading public and private institutions, has fully implemented Patient Blood Management supported by national institutions including the National Blood Authority (NBA), the Australian Commission on Safety and Quality in Healthcare, the Western Australia Department of Health, and the Australian Red Cross Blood Service. In South Korea, Patient Blood Management was implemented in few institutions about a decade ago, followed by a broader strategic approach supported by national authorities. In China, Turkey and Mexico, Patient Blood Management implementation originated with leading clinicians (“champions”) of large national institutions and is now increasingly recognized by the authorities. In South Africa, the implementation of Patient Blood Management is led by the South African National Blood Service and supported by a national Patient Blood Management expert group [21].

**Table 2:** Summary of interview responses to the questions relating to the current status and approach to implementing Patient Blood Management on the national or local level

	Australia	Brazil	China	Croatia	Greece	Lebanon	Mexico	Saudi Arabia	South Africa	South Korea	Switzerland	Turkey
	AUS	BRA	CHN	HRV	GRE	LBN	MEX	SAU	ZAF	KOR	CHE	TUR
**Stage**	MATURE; National Policy; Broad uptake	INITIATION; Scattered individual leadership	ADVANCED; National Drive	INITIATION; Specialist society drive	INITIATION; National drive through BB	INITIATION; individual initiatives	INITIATION; national project and coordinator	INITIATION; in some hospitals / institutions	INITIATION; individual initiatives.	ADVANCED; implementa-tion project; strong PBM society	INITIATION / ADVANCED; in some hospitals / institutions	ADVANCED; Broad awareness through cross- specialty implementation project
**Approach**												
**Top Down**	NBA; States Legal framework, standards & guidance Quality certification	N.A.	National Health Commission, PBM as part of blood supply & demand strategy	MoH informed Some coordination by specialist society (network)	National BB (with MoH) and in cooperation with hospital managers	Original political drive suspended due to political situation	National project with Quality certification	National interest group, but not much coordination	N.A.	National drive: collaboration program for pilots, data collection, and setting standards	Limited due to strong federal healthcare policy structure	EU Project for PBM implement-tation
**Bottom up**	Initially driven by individual leadership and multi-disciplinary teams in hospitals	Individual Leadership	Clinical champions in leading pilot hospitals	Clinical/ de-partmental champions	Individual initiatives in some hospitals / institutions	Individual initiatives in some hospitals / institutions	Coordinated bottom up	Introduced in some hospitals / institutions	Individual initiatives	Individual initiatives in single hospitals over past 10 years	Strong in a few leading hospitals based on individual leadership	Leading institutions, broad awareness through specialist societies

‘Mature’ describes a high level of implementation; ‘Advanced’ denotes a strategic, coordinated approach towards general implementation; ‘Initiation’ describes the occurrence of few individual initiatives with low level of coordination

*Abbreviations*: *BB* Blood Bank, *EU* European Union, *MoH* Ministry of Health, *N.A.* Not available, *NBA* National Blood Authority, *PBM* Patient Blood Management

Croatia, Greece and Lebanon seek the dual pathway, although the current political situation in Lebanon has put all governmental support to a halt. Brazil, Saudi Arabia, and Switzerland currently rely on local clinician-led initiatives (bottom-up).

### Drivers for the implementation of patient blood management (question 7A)

**Fig. 1:**
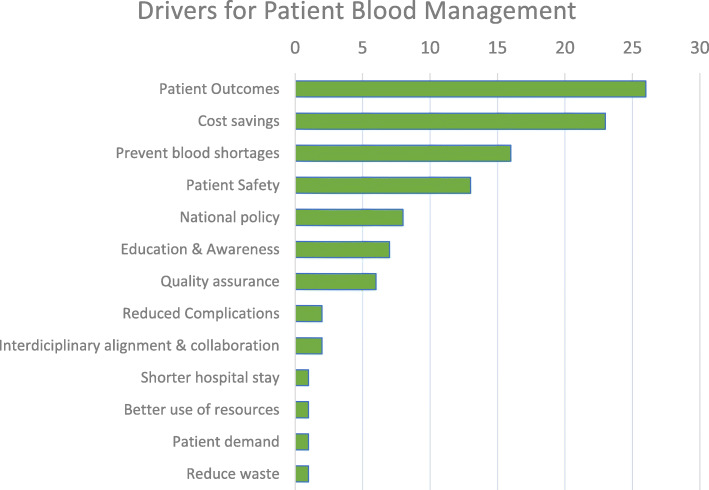
Drivers for Patient Blood Management. Quantitative evaluation of all drivers mentioned by the interviewees when asked the question” What could be the main drivers for Patient Blood Management – Why is Patient Blood Management needed?”. The responses were spontaneous and unprompted. (*N* = 46) The driving factors were sorted by the total number of mentions (top to bottom decreasing). Note: The number of mentions is not a measure for the strength of a specific driver. Education & Awareness is abbreviated for ‘education and awareness relating to the risks of transfusion and the benefits of Patient Blood Management’

Of the 11 drivers mentioned unprompted during the interviews (Fig. 1), patient outcomes (26 mentions), cost savings (23 mentions), preventing or better dealing with blood shortages (16 mentions from Sth. Korea, Turkey, Mexico, China, Brazil), improving patient safety or reducing complications (15 mentions from Brazil, China, Sth. Korea, Saudi Arabia, Turkey) were quoted most frequently. Several experts also mentioned national policy [8], education and awareness (concerning the risks of transfusion and benefits of Patient Blood Management) [7], and a quality assurance system [6].

*Shorter length of hospital stays, better use of resources,* and *reduction of waste* were only mentioned once each. P*atient demand* was considered to become a driver once the risks related to transfusion and the benefits Patient Blood Management were recognized more broadly in the general population.

### Barriers for the Implementation of patient blood management (question 6)

Except for Australia, where Patient Blood Management is already widely adopted into practice, the need to *change work practice* was rated as the most prominent barrier for the implementation of Patient Blood Management as shown in Table 3. The need for *collaboration and communication* was rated equally important across the countries, followed by the *lack of experience with Patient Blood Management, the feasibility to integrate Patient Blood Management into the current processes*, and *strong belief in transfusion.*

**Table 3:**
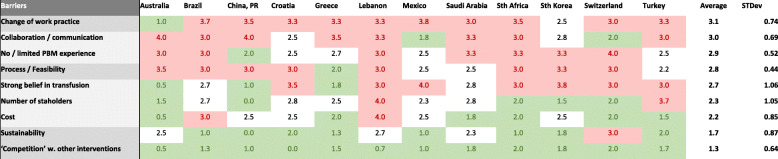
Rating of Barriers for Implementation by perceived severity of the barrier. The rating was between 0 (is no barrier) and 4 (high barrier). The barriers were sorted by the average rating (top to bottom decreasing). (*N* = 35) Color coding: red for average country values of 3 or higher, white for values between 2.01 and 2.99, and green for values of 2 or lower

### Accelerators and inhibitors for the implementation of patient blood management (question 7E)

**Fig. 2:**
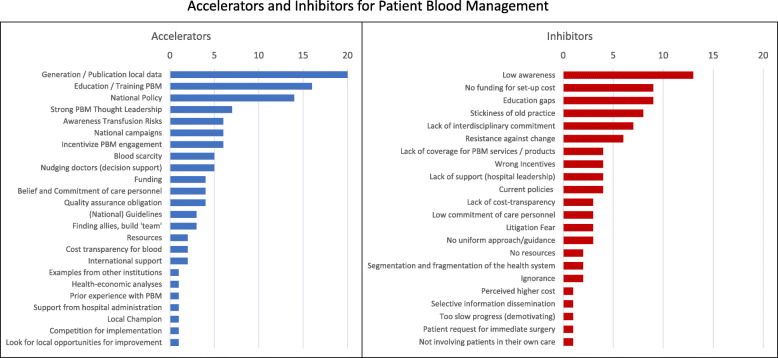
Accelerators and Inhibitors for Patient Blood Management. Quantitative evaluation of factors mentioned by the interviewees when asked for the factors which would accelerate (Accelerators) or delay or inhibit (Inhibitors) the implementation of Patient Blood Management from their perspective. The responses were spontaneous and unprompted. (*N* = 46) The items were sorted by the total number of mentions (top to bottom decreasing). Note: The number of mentions is not a measure for the strength of a specific accelerator or inhibitor

The responses for factors accelerating or supporting Patient Blood Management implementation fell into 24 categories as shown in Fig. 2*(left part)*. *Generation of local data and evidence*, *education and training for Patient Blood Management,* a *national Patient Blood Management policy*, *and strong thought leadership*, were the most frequently mentioned factors. *Blood scarcity*, *funding*, *awareness of transfusion risks*, *incentives for Patient Blood Management engagement*, *belief and commitment of care personnel*, and *quality assurance obligation* were also frequently mentioned. During the final six interviews between February and May 2020, the COVID-19 pandemic was newly mentioned as potential accelerator due to increased blood scarcity and potential blood safety issues.

The inhibitors or delaying factors fell into 22 categories (see Fig. 2*, right part)* with the most frequently mentioned being low awareness, no funding for set-up cost, education gaps, and stickiness of the old practice (even stronger if combined with the responses for the closely related *resistance against change*), *lack of interdisciplinary commitment*, and *resistance against change*.

### Stakeholders (question 7B)

**Fig. 3:**
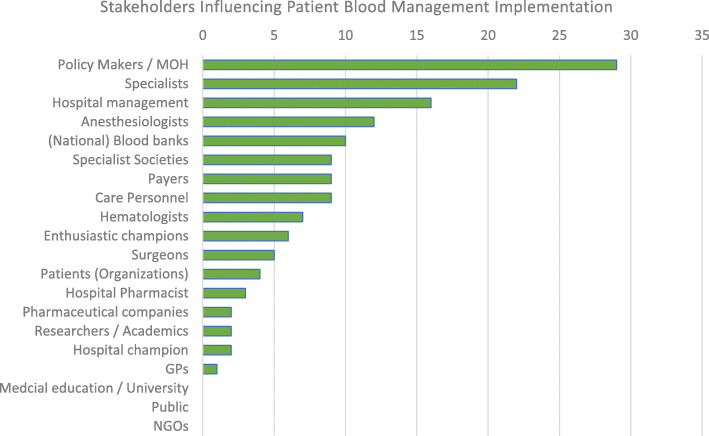
Stakeholders Influencing Patient Blood Management Implementation. Quantitative evaluation of categories mentioned by the interviewees to the question “Who in your opinion will be the essential stakeholders who will have to be involved / convinced?”. The responses were spontaneous and unprompted. (*N* = 46) The stakeholder types were sorted by the total number of mentions (top to bottom decreasing). Note: The number of mentions is not a measure for the importance of a specific stakeholder. Medical education, Non-governmental organization (NGO) and the Public were not mentioned as important stakeholders specifically, but they were mentioned in other parts of the interview as important groups and therefore were added for completeness

Sixty-three percent of the interviewees (29 of 46) identified policy makers (National Health Council, Ministry of Health, etc.) as important stakeholders in Patient Blood Management implementation. As shown in Fig. 3, the majority also listed either specialists in general [22], or specific specialists (12 x anesthesiologists, 7 x hematologists, 5 x surgeons), 35% (16 of 46) included the hospital management. Other stakeholders (professional societies, national or regional blood banks, payers, nursing staff, enthusiastic champions, hospital pharmacists, patients/patient organizations, pharmaceutical companies, researchers/academics, hospital champion, general practitioners were mentioned less frequently or only in other parts of the interview (medical schools, non-governmental organizations, or the public at large).

### Coding and clustering of implementation measures

After translating accelerators and inhibitors into actionable measures and clustering these measures by the type (level) of intervention, six levels for intervention were identified: government/policy, funding, research, healthcare providers, education/training, and public/patients. On each of the six levels specific measures can contribute to the implementation of Patient Blood Management as reflected in Table 4 with reference to the concrete examples reported by the implementors.

**Table 4:** Summary of measures to consider for implementing PBM, sorted by level of intervention: Government (national and/or regional), funding, research, healthcare provision, training/education, and patients

Measures	Rational / Expectations	Examples	Points to consider
**GOVERNMENT LEVEL (national or regional)**			
National Policy	• National initiatives and guidance push the hospitals towards PBM implementation	• AUS: National policy for PBM and national measures to support implementation • TUR, KOR: Close collaboration of PBM leaders with MoH • TUR: qualification for a 3-yr EU grant dedicated to a systematic country-wide implementation of PBM [55] • CHN: officially addressed the importance of PBM to improve clinical practice [56]	CHALLENGES • Changing of policy priorities; political instability (LBN) • Policy priority of shifting from tertiary hospitals to primary care level antagonizes the pre-operative PBM interventions (CHN) • National policy not a game changer in countries with decentralized healthcare (CHE)
Blood Shortage	• Actual and anticipated blood shortage is recognized on a policy level and requires action; donation volume is shrinking, the demand for blood is increasing (aging population) • Donor deferrals due to new or re-emerging pathogens; cancelled blood collections due to lockdown during epidemics • Family replacement schemes: mandatory donations may increase risk and limit access	• ZAF, CHN, MEX: Frequent supply issues • HRV, GRE: Seasonal shortages • KOR, ZAF: Shortage and COVID-19 virus risk^a^ • BRA: Zika-Virus [57]; supply issues in public system • AUS, TUR: Shortage predicted • LBN, GRE, MEX: Replacement modus • CHN: 30% Reciprocal blood donation	REMARK • Impact of COVID-19 pandemic on blood supply [58]
Strong PBM Thought Leadership	• Fosters a broader country-wide acceptance and change • Liaise on policy level, engage with payers, engage specialist societies, and introduce medical curricula	• KOR: Korean PBM Society with multi-disciplinary leadership function • TUR: EU-funded project for PBM implementation across Turkey	REMARK • May be difficult for few individuals to cover that scope and thus, formation of a high-level work or interest group may be advisable
PBM Incentives	• Attract clinicians to become part of the change • Increase level of experience and familiarity with PBM	• CRO: participation in international clinical study • TUR, KOR, MEX, ZAF: National pilots & research opportunities	REMARKS • Involve practitioners actively in research • Recognition of individual initiative through active engagement and authorship
National Guidelines	• Adapting international guidelines to local healthcare context can be essential for acceptance • National guidance will facilitate coordinated and homogeneous activities across the country	• ZAF, TUR, KOR, MEX, BRA, HRV, CHN: ongoing projects to locally adapt international guidelines	RISK • Scattered / fragmented approaches will make it difficult to consolidate in best practice
**FUNDING LEVEL**			
Alignment of policy and funding	• Consensus for a reimbursement and funding solution	• KOR: Center for Disease Control in the MoH and the Health Insurance and Reimbursement Agency (HIRA) committed to PBM related projects (1) auditing the current level of transfusion appropriateness in each hospital, and (2) funding dedicated projects on PBM implementation in the country	CHALLENGE • Heterogeneity in access to healthcare and its funding requires different approaches for funding and reimbursement of PBM (MEX, LBN)
Reimbursement	• Increase the willingness to invest in establishing PBM • Adjust reimbursement systems to incentivize improved health outcomes and efficiency and disincentivize transfusion volume [59, 60].	• KOR, MEX, AUS, TUR: Funding national pilot or full implementation projects • BRA: volume-dependent reimbursement to hospitals (fee for service) incentivizes a high use of transfusions; but first examples of capitation-based hospital reimbursement emerge (supportive for PBM)	CHALLENGES • Potential other sources of funding (NGOs, special international projects) • Funding always compromised during (economic) crises
Cost transparency for blood; Cost ‘fairness’	• Mandate full cost transparency of transfusion and PBM to allow for cost-effective allocation of (public) funds	• GRE, HRV: Not knowing the cost of blood products or artificially low cost impedes adoption of PBM	REMARK • Even if at zero cost to the hospital, blood products are not for free from a societal perspective
Funding and resources in hospital	• Secure funding necessary for setting up the infrastructure (including point-of-care testing devices, cell salvage equipment, pre-operative anemia clinic, continuous medical education (CME) and training • Identify and remove dis-incentives	• HRV, GRE, LBN, MEX: Difficulties in securing funding despite principal support for the concept • BRA, LBN: Fee for service dis-incentivizes PBM (imbalance between profitability and patient health) • Alignment across budgets: e.g. pharmacy budget vs other cost	REMARK • Use measures / local data to demonstrate the realistic budget needs, ROI, time frame required [61].
**RESEARCH LEVEL**			
Quality measurement/assurance	• Use of quality measures, to track blood use (i.e., units ordered, used, and discarded per hospital, ward, type of intervention and individual specialists) to shift focus to patient needs and outcomes	• KOR, MEX, CHE, AUS: pursuing quality and performance measurement initiatives	REMARK • Performance measures empowers local transfusion committees and PBM implementation task forces
Collecting and publishing local data	• Demonstrating impact of PBM with local data on clinical outcomes, adverse events or complications, • Capturing and reporting local epidemiology data (prevalence) • Quantify opportunities, risks, and cost for PBM in the local setting; ideally as multi-disciplinary intra- or inter-hospital collaboration	• AUS, CHE, KOR: local data collection systems initiated or established to enable reporting, benchmarking, or performance analysis • TUR: publication of local data [62]	REMARKS • Local evidence helps to refute that the international experience may not be transferrable to the local context • Local research motivates participants to gain expertise and to become part of the change
Health-economic analyses	• To convince stakeholders of the cost-effectiveness of PBM, analyses must be based on local data (cost / outcomes)	• Health-economic evidence from AUS, CHE, GER, and the USA [63, 64]	CHALLENGE • Current H/E evidence from countries with specific economic and health-economic settings and may not be generalizable
International support and collaboration	• Cross-fertilize and share the learnings transnationally	• International collaboration is frequent, e.g. strong engagement of IFPBM & SABM, ZAF w. National Blood Authority in AUS, KOR w. AUS, BRA w. SABM.	REMARK • Includes international teaching, web-based services, advisory exchange, or involvement of experts in another country’s task forces.
**HEALTHCARE PROVIDER LEVEL**			
Communication	• Strengthen belief and commitment of clinical staff • Re-align all stakeholders around the transfusion process	• GRE: Generation of an intra-hospital consensus and protocol with reporting system for restrictive blood use • MEX, ZAF, AUS: continuous communication, involvement, and feedback by coordinator / initiator in hospital, • ZAF, MEX: Chat-group in a social media platform to report local experiences, announce events, and post relevant publications, questions. and suggestions	A common vision and buy-in by those who need to change their practice is essential to achieve change [65]
Identify allies, build teams	• To increase clout and trust across specialties • Foster multi-disciplinary collaboration, mutual endorsement and support	• LBN: Expanding across specialties already in initial phase added great impetus MEX, TUR: Multidisciplinary PBM Academies; LEB, KOR, ZAF: Multidisciplinary Iron Academies	REMARKS • PBM is a team effort [15, 21, 61] • Supports forming a guiding coalition [65]
Prior experience with PBM	• Expand the knowledge and openness for PBM by involving care personnel from different disciplines in implementation projects	• Pilot projects in several hospitals/wards to involve and expose them to PBM methods	REMARK • Overcome the stickiness of the old practice [66] and resistance to change
Ensure support from hospital administration	• Design/align the organization to enable optimal and sustainable PBM across specialties • Secure funding for staff, systems support (IT), other resources • Get approval to establish a multi-disciplinary PBM committee	• Most initiatives reported that alignment with hospital administration / CEO was improving chances for success • HRV, GRE: Activities under departmental responsibility may not need agreement by hospital management. • LBN, SAU, HRV, LBN: To get funding for establishing PBM was difficult and therefore done within the existing resources (overtime) • BRA, MEX: dedicated project management ensures planning and roll out across specialties / departments	REMARKS • While small changes could be introduced individually or within one specialty the full potential can only be achieved with multi-disciplinary change • Understand the economic and system incentives and to be in close communication to collaboratively identify the path to implementation (milestones, tasks, and responsibilities)
Local champion (Medical Director or project coordinator for PBM)	• Responsible for planning, organizing and directing PBM, supporting specialists, and ensuring continuous data collection, reporting and benchmarking,	• HRV, GRE, MEX: general role in training, education, information, protocol development • BRA: Change management • AUS, CHE: organize PBM at patient level (case management)	REMARK • PBM coordinator can be a success factor for sustainability (AUS, CHE)
Hospital protocols (SOPs)	• Tailor PBM protocols to the specific hospital context and routines • Increase local ownership across the disciplines, interdisciplinary commitment	• HRV, GRE, TUR, MEX, BRA: Several interviewees reported the development of local protocols before the availability of National Guidelines	
Data collection, reporting & benchmarking system	• Shows impact, measures gaps, and helps to improve quality of care	• ZAF, KOR: currently developing a monitoring system in hospital(s)	
Nudging clinicians & stimulating competition	• Using IT or quality reporting systems to motivate and remind physicians to practice PBM • Using the competitive nature of people to motivate them to excel in PBM	• AUS, CHF, MEX, ZAF reported use or plan to use competitive forces or ‘nudging instruments’ to remind practitioners to improve their PBM practices (reminders, league tables)	REMARKS • Include IT and/or quality specialists in developing the local procedures for mapping into data collection and analytical support tools • Nudging = nonregulatory and nonmonetary interventions that steer people in a particular direction while preserving their freedom of choice” [67, 68]
Involving the entire care team	• Alignment, participatory processes	• ZAF: Importance of involving nurses who have high influence on the patient care • GRE: Importance of aligning the ordering of blood products.	REMARK • Includes nursing, hospital pharmacy, blood ordering process to ensure common goals
Seizing local opportunities for improvement	• Create momentum: Use opportunities in own environment for starting with specific aspects of PBM • Move forward faster and prove success	• HRV, LBN: Start within ward/ department • ZAF: start with communication & education of hospital specialists • MEX, BRA: pilots	REMARK • Even small ‘wins’ will motivate people
**TRAINING & EDUCATION LEVEL**			
Education and Training for PBM	• Identify and address knowledge gaps among specialists • Update under-and postgraduate curricula	• AUS: Integration in medical school (University of Western Australia) curriculum & exams • MEX, ZAF, TUR: PBM academies and/or continued medical education (CME) for practitioners • AUS, ZAF: online training material [69]	REMARKS • Training of all specialists concerned (incl. anesthesiologist, intensive care specialists, surgeons, hematologists, oncologists, gastroenterologists, obstetricians & gynecologists) and nursing staff in relation to benefits of PBM, • Avoid asymmetry in information to prevent that ‘eminence wins over evidence’ in the choice of therapy
Increase Awareness Transfusion Risks	• Overcome eminence-based practice (“transfusion is always beneficial”) and increase the knowledge about the associated risks	• Global: Many of the specialists who administer transfusions during surgery (surgeons, anesthesiologists) often don’t see the mid- or long-term complications (infections, immune reactions, thrombosis).	REMARKS • Necessitates re-education of all participants in the transfusion decision • Requires information, education, and reminders across specialties (publications and newsletters, conferences, social media-channels)
Medico-legal aspects and protective measures as part of PBM training	• Strengthen the assertiveness of physicians relating to PBM	• BRA: Litigation is commonly used by patients to get access to procedures which they perceive to be beneficial	
**PATIENT & PUBLIC LEVEL**			
National information campaigns	• Develop awareness for PBM • Encourage patients to discuss PBM at their doctor’s appointment • Prevent litigation against physicians following guideline-compliant restrictive transfusion strategies • Decrease patient demand blood transfusion	• KOR, LBN, ZAF: Initiated or conducted national awareness campaigns through important media channels • BRA: Litigation is commonly used by patients to get access to procedures which they perceive to be beneficial	RISKS • If done too early, doctors might be overwhelmed by patient demand • Too much information on transfusion risks may negatively impact the willingness of the public to donate blood REMARK • Involving patients, collaborating with patients, and informing the public may improve understanding and reduce the risk for litigation
PAG initiatives	• Co-create national information campaigns (PBM thought leaders, politicians, PAGs) • Explore patient experiences and preferences • Engagement / advocating for PBM insurance coverage • Achieve comprehensive patient education on risks and benefits of all treatment options (including transfusion) for anaemia, blood loss and coagulopathy • Ensure fully informed consent and/or shared decision making • PAGs to request PBM certification and/or hospitals accreditation	• HRV, KOR: Initial contacts • TUR: In contact with 5 NGO’s, who receive regular information • GRE, LBN, HRV, KOR, BRA: increasing demand for participatory medicine and shared decision making by PAGs and/or healthcare policy	REMARKS • PAG-patient interaction relating to transfusion and/or PBM not yet common • Co-creation / co-production: researchers, practitioners and the public join efforts and share responsibilities to develop, implement, monitor, evaluate and re-develop interventions [70]

*Abbreviations*: *MoH* Ministry of Health, *SABM*, https://sabm.org Society for the Advancement of Blood Management, *ROI* Return on Investment. *Country Abbreviations*: *AUS* Australia, *BRA* Brazil, *CHN* Peoples Republic of China, *HRV* Croatia, *GRC* Greece, *KOR* Republic of Korea, *LBN* Lebanon, *MEX* Mexico, *ZAF* South Africa, *CHE* Switzerland, *TUR* Turkey, *PAG* Patient Advocacy Group, *IFPBM* International Foundation Patient Blood Management, *SABM* Society for the Advancement of Blood Management

^a^The risk of COVID-19 viral infection only became apparent starting in January 2020. Hence, this threat was only mentioned in the last interviews (KOR, ZAF, SAU)

## Discussion

### The challenge

**Fig. 4:**
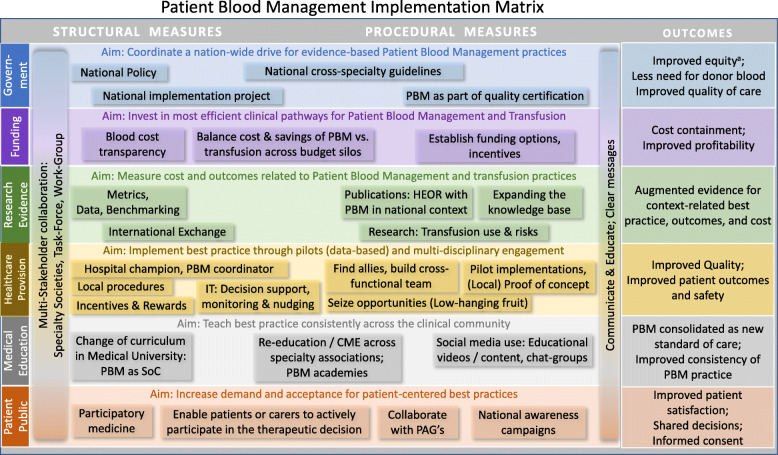
Patient Blood Management Implementation Matrix. Implementation matrix summarizing the aims, measures, and expected outcomes of comprehensive Patient Blood Management across six implementation levels. This implementation matrix is derived from the full table of measures (Table 4). ^a)^ Equity: access to evidence-based blood preservation for all patients/citizens in the country

Unless translated into the daily routine and organizational culture, evidence is of limited value [71]. To bridge the gap and effectuate the necessary culture change, it is essential to understand the drivers and barriers for Patient Blood Management as well as the stakeholders’ roles and responsibilities. An essential challenge in replacing the long-standing, well-organized, product-centered culture of transfusion medicine by the patient-centered model of Patient Blood Management is that most diverse stakeholders need to communicate, collaborate and overcome the complexity of the Patient Blood Management implementation process. This starts with their specific contribution to the systemic implementation as summarized into the implementation matrix displayed in Fig. 4, which was derived from the full and detailed collection of measures identified from the interviews (Table 4*).* We will discuss each level of the table in more detail in the passages following below.

### Using the Implementation matrix to develop patient blood management strategies

The Patient Blood Management-implementation matrix, as derived from the interviews, guides Patient Blood Management implementors in systematically identifying effective measures for Patient Blood Management implementation depending on the economic and healthcare context in their country.

These measures will be discussed in more detail along six implementation levels and consolidated in the final section into a Guided Implementation recommendation.

### Government level

Patient Blood Management is expected to improve quality of care, reduce dependency on donor blood, and contribute to better access to healthcare and equity (evidence-based blood preservation for all patients/citizens in the country). In Western Australia, hospital stays were reduced by almost 70,000 days over 5 years [15]. Suchlike improvement enhances capacity of care and consequently, patient access, and resource utilization. Likewise, the savings due to Patient Blood Management allow for better allocation of scarce resources, thus increasing productivity of the healthcare sector. This should motivate national policy makers to prioritize Patient Blood Management.

National policy makers and senior representatives of the Health Ministry are important stakeholders in coordinating Patient Blood Management implementation nationally (see Fig. 3). Reporting and incentivization of key performance indicators, accreditation of healthcare providers for Patient Blood Management, Patient Blood Management certification of clinicians, and funding and facilitating the development of multi-disciplinary national Patient Blood Management guidelines form essential structural elements for driving Patient Blood Management implementation nation-wide.

However, structural changes on government level usually require long time. One implementor stated “it takes more than seven years to introduce a policy in our country”. Creating a sense of urgency through multiple stimuli, including success stories demonstrated in pilots and the generation, publication, or communication of the evidence, can help to overcome the inertness for introducing a new medical model perceived as being complex [10].

### Healthcare provider level

Patient Blood Management offers the rare opportunity to improve patient outcomes while reducing resource utilization and cost [15, 72, 73]. The healthcare provider related measures reported by the implementors start with the identification of local champions and allies from clinical and non-clinical departments to create the sufficient momentum and mass for the implementation. The securing of funding, information technology (IT) infrastructure and support to enable Patient Blood Management data collection, reporting and benchmarking was deemed equally necessary as establishing multi-professional teams, Patient Blood Management committees, program coordinators and nurses. As recommended previously by others [19, 61] and aligned with recognized approaches to change [74, 75], implementors preferred a piloting approach (“harvest low hanging fruit”) accompanied by the development of internal capability, aiming to gain practical experience and to optimize the Patient Blood Management processes in the local context. Other important modules on the provider level were developing Patient Blood Management standard operating procedures, defining key performance indicators, and measuring outcomes.

Electronic clinical decision support systems for controlling transfusions were deemed effective, also if combined with systems to incentivize and reward the progression towards Patient Blood Management. Electronic transfusion decision support systems can effectively reduce transfusion rate and index in the daily routine [76, 77] and serve as a ‘nudging’ mechanism. ‘Nudging’ denotes “non-regulatory and non-monetary interventions for changing behavior that steer people in a particular direction while preserving their freedom of choice” [67, 68]. This includes automated or targeted reminders, individual performance reviews based on local data collection and analysis, or Patient Blood Management dashboards as reported elsewhere [78].

### Training and education level

To avoid asymmetry of information and conflicting behaviors within the hospital, training, and communication on Patient Blood Management needs to address the entire clinical staff including clinical specialists, nurses, pharmacists, and others influencing decisions related to managing patients’ blood. Implementors suggested that clinical knowledge and skills for Patient Blood Management must be embedded in both under- and postgraduate education (curricula in medical schools, accredited continuous medical education, Patient Blood Management academies, and e-learning- and information-platforms). However, except for Western Australia, Patient Blood Management is currently not part of the undergraduate curriculum of medical students. Like Patient Blood Management preceptorships, educational and training activities for Patient Blood Management are currently organized for post-graduates, often initiated by the implementors and local Patient Blood Management champions, and mostly industry sponsored. Implementors should liaise with the leadership of academia and medical schools to firmly integrate Patient Blood Management into the undergraduate education in alignment with the federal Ministries of Health and Education, where applicable.

### Research level

Patient Blood Management offers a broad spectrum of new experimental, clinical, epidemiological, and health-economic research opportunities, as evidenced by the growing number of research publications. Benchmarking and reporting of key performance indicators for Patient Blood Management yield valuable insights concerning clinical and economic outcomes related to Patient Blood Management. Further research as well as national and international exchange will help to improve Patient Blood Management techniques as also highlighted by international thought leaders [29, 61, 78, 79]. Most importantly, as an essential prerequisite, the implementors demanded to generate and communicate local evidence (prove of outcomes and cost-effectiveness in the local context at local cost structures) to link the implementation across hospitals and to foster policies on the national level.

### Funder level

Public funders may benefit from Patient Blood Management through reduced average length of hospital stay and lower resource consumption, resulting in cost containment and better resource use. Private funders may expect higher profitability, in particular with diagnosis related groups (DRG) or value-based reimbursement systems (e.g., accountable care): in DRGs with high anemia prevalence and potentially high blood loss such as obstetrics, cardiovascular surgery or oncology, the total cost per episode of care have shown to decrease over time, thus leading to reduced tariffs [80]. For Germany, overall yearly cost-savings with elective surgery were calculated to be €1029 million - almost 1.58% of the total national hospital budget [81].

Even in fee-for-service settings, funders may benefit from Patient Blood Management: currently, they might reimburse hospitals for the number of transfusions administered, while patients pay for their anemia treatment out-of-pocket. Where transparent, implementors in the interviews reported increasing cost of blood components (per unit) due to increasing measures for quality and safety testing. Once funders begin incentivizing (pre-operative) anemia management as an essential part of Patient Blood Management, they foster better outcomes, fewer complications, and shorter hospital stays, thus reducing the overall reimbursement cost per episode of care as compared to the currently established transfusion preferences [15, 63]. The cost of quality assurance and administering these blood products is a multifold of the actual acquisition cost and therefore, represents a substantial cost volume for the hospital and consequently for the funder, even where allogeneic blood products are covered by national funds and are considered ‘free’ [82, 83].

Appropriate reimbursement of Patient Blood Management including anemia management was a strong request in our interviews, and implementors even proposed to incentivize Patient Blood Management for healthcare providers. Given the documented savings potential with Patient Blood Management [15, 64, 81, 84–86], it should be a priority for implementors to inform, educate and engage funders on this important issue. Following the example of the German health insurance BARMER [80], insurers may even help underpinning the Patient Blood Management value using their own data to demonstrate savings with improved outcomes.

### Patient level

According to the implementors, Patient Blood Management and its benefits are largely unknown to patients, despite being the ‘big winners’ from Patient Blood Management with significantly improved clinical outcomes, safety, and reduced average length of hospital stay. Patients usually seek medical treatment based on a proper diagnosis and expect to be treated with safe and effective medical or surgical interventions. Unless being informed by their treating physician and being involved for shared decision making, they would not know that Patient Blood Management improves their chances for earlier discharge from hospital and reduces their risk for hospital acquired infection or even mortality. Patient advocates could contribute by creating Patient Blood Management awareness, but also by educating for and defending patients’ rights. Collaborating and likewise, supporting national campaigns to emphasize safety and the beneficial outcomes of Patient Blood Management, could foster shared clinical decision making and informed consent. Some implementors even saw the potential for patient advocates to approach funders to incentivize and support Patient Blood Management.

Potential risks were expected by one implementor when entering the public domain too early and thus, creating demand before physicians would be sufficiently familiar with Patient Blood Management and its benefit. Another implementor cautioned, that too much information on transfusion risks may negatively impact on the willingness to donate blood. Involvement of patients or patient advocates should be planned thoroughly within the country culture and context. However, the aim to *involve patients more in their own care* [87], the strive for *‘person-centered healthcare’* [88], and the priority of increased patient safety [89–91] conforms to physicians’ obligations towards educating and informing patients about all risks and benefits of available treatment options. Medico-legal experts increasingly caution that widespread disregard of transfusion associated risks for adverse outcomes may result in litigation against those neglecting physicians and specialists [92]. Informing the public and the patients in collaboration with patient advocacy groups can be a powerful element of the Patient Blood Management implementation strategy. Engaging the public and patients will not only result in more demand for Patient Blood Management but also improve patient satisfaction and foster participatory medicine.

### Guided Implementation

In some of the countries described in this survey, Patient Blood Management was implemented simultaneously from bottom-up (e.g., from a department level or hospital/clinical level) and top-down (driven by policy and/or hospital administrative leadership) (see Table 2) with large variation in the closeness of the interaction between policy and operational levels. In other countries, implementation progresses just through the bottom-up pathway, predominantly initiated, and led by individuals or small groups with different clinical background or innovation managers. To effectively coordinate and execute a statewide or even national implementation project across all six interdependent layers requires governance [15, 20, 93]. Following the example of Western Australia [15, 93], the EU Guide for Health Authorities [20] suggests that National Patient Blood Management Steering Committees, preferably under the authority of the Health Ministry, should coordinate planning and provisioning of Patient Blood Management resources, structural requirements, and national and international Patient Blood Management research efforts. Transitional tasks forces were proposed to develop national Patient Blood Management reimbursement schemes and managing Patient Blood Management transition costs (i.e. costs to manage the ‘paradigm shift’). Likewise, National Patient Blood Management Steering Committees could facilitate broad and homogeneous adoption, supported by national programs or committees for guidelines development, data collection, benchmarking, and analytics.

The experiences and expectations of the implementors confirmed the importance of adapting to the local healthcare and cultural context and aligning with the local / national healthcare priorities and funding situation. Implementation success depends on good change strategies. Resistance from the old transfusion paradigm due to ignorance and conflicting incentives needs to be overcome, and the Patient Blood Management paradigm must be anchored in the healthcare delivery culture. Future research, for example with proponents of the transfusion priority, could further investigate the motivation for such resistance and possible ways to overcome it.

Kotter’s model for managing change embraces eight essential accelerators: establishing a sense of urgency, creating a guiding coalition, developing a change vision, communicating the vision for buy-in, empowering broad-based action, generating short-term wins, never letting up, and incorporating changes into the culture [65, 94]. This should be considered regardless of where implementation starts, whether on a national, regional or hospital level, or initiated by a blood bank. Concurrent action, well adapted to the local context, across all eight change accelerators while rapidly building a network of change agents should maximize its adoption and impact [65]. For example, for a clinician it is probably easiest to start the process in the own specialty, which is within her or his own control. However, even at that stage, it would be good to design an implementation plan aiming for broader implementation. An example for a stepwise implementation starting on the hospital level is available in the online material as a part of a slide show summarizing the key findings of this manuscript (Additional File 3, slide 8). Of course, careful planning the implementation is indispensable when aiming at wider adoption of Patient Blood Management.


**Additional file 3.**


The implementation matrix (Fig. 4) may serve as a guidance in planning, even if starting with a small pilot. Following Realist Evaluation approach [95] the user should see the matrix as a ‘menu’ of elements for designing the local implementation. On each level, the implementors need to assess what works (best) in the local context, when, and which stakeholders should be involved for successfully creating the implementation path for Patient Blood Management in the country or organization.

Adaptation to the local context depends on access to and inclusion of the key stakeholders and influencers within the own healthcare environment. Implementors need to identify the stakeholders in implementing Patient Blood Management and understand what motivates each of them to support, engage, or contribute [19–21].

## Limitations

The following limitations should be considered for this research. The selected countries cannot be fully representative for all countries and healthcare systems across the world. However, they were from five continents and included healthcare systems of high or lower income, and the interviewees were professionals leading and/or promoting Patient Blood Management in the healthcare sector of their respective environment. The latter may also be a limitation of the study as we did not interview stakeholders who are opposing the adoption of Patient Blood Management. A survey with these groups could help to better understand and address potential resistance or opposition. The impact of the various implementation measures across six levels could not be determined. Once Patient Blood Management will be established in more countries and healthcare systems, key performance indicators might be linked to specific measures and rated, thus showing their relative importance.

## Conclusion

With the objective of learning from the practical experiences with the implementation of Patient Blood Management, structured interviews were conducted with a multi-disciplinary group of Patient Blood Management implementors in 12 countries reflecting initial, advanced, and full level of implementation. There was consensus that patients would benefit most from Patient Blood Management, with improved outcomes including morbidity, mortality, quality of life, average length of hospital stay, and patient safety. The expected improvements in outcomes and cost savings as well as more efficient use of (blood) resources were identified as the core drivers. The need for *changing work practice* and for *collaboration and communication* and the *lack of experience with Patient Blood Management* were rated as most important barriers for Patient Blood Management. After converting the identified accelerators and inhibitors for Patient Blood Management into actionable implementation measures, six levels for intervention were identified, including government, healthcare providers, education, funders, research, and patients. This forms the framework for a six-level implementation matrix, describing all measures and expected outcomes as reported by the implementors.

## Supplementary Information

**Additional file 1.**

**Additional file 2.**

## Data Availability

The results are presented in aggregated form. The original data are accessible through the corresponding author on reasonable request. The interview questionnaire is available as additional online material (Additional File 1).

## Abbreviations

AUS: Australia
BRA: Brazil
CHN: Peoples Republic of China
HRV: Croatia
GRC: Greece
KOR: Republic of Korea
LBN: Lebanon
MEX: Mexico
SAU: Kingdom of Saudi Arabia
ZAF: South Africa
CHE: Switzerland
TUR: Turkey
CME: Continuing medical education
GP: General practitioner
LMIC: Low- and middle-income country
MoH: Ministry of Health
PBM: Patient blood management
QoL: Quality of life
SoC: Standard of care
WHO: World Health Organization
