# Supplementary material for: Making patient blood management the new norm(al) as experienced by implementors in diverse countries
Source: BMC Health Serv Res. 2021 Jul 2;21:634. doi: 10.1186/s12913-021-06484-3 (PMC8249439; doi:10.1186/s12913-021-06484-3)
Supplement: Supplementary file 2 — Additional file 1. [file 12913_2021_6484_MOESM1_ESM.docx]

Introducing Patient Blood Management (PBM) as Standard of Care

Interview Guide

| Name: |  |
| --- | --- |
| Organisation: |  |
| Function: |  |

| **Professional education:**  Medical doctor  Pharmacist  Business / Finance  Nursing  Sciences / Research  Health-economist  Patient advocate  Other: _____________ | **Working at**  Hospital: _________  Blood Bank  Research  Ministry of Health  Insurance  Industry  Academics    Other: _____________ |
| --- | --- |

We will record this interview for our own documentation and to enable us to confirm your answers in case of uncertainty. The recordings will not be published. We may cite specific wordings but only with your consent.

**SECTION 1**

1. How would you define Patient Blood Management?
2. Can you please describe your experience with / in relation to PBM?
3. What is the current status of introducing the PBM policy in your country / your organization?
   - Is there a National PBM policy?
     1. **If yes**, please describe
     2. **If no**, is there a national policy planned / anticipated?
   - What is the level of adoption of PBM in the country? Is PBM implemented in any or some hospitals?
     1. If yes, please describe
     2. Is PBM practiced in some hospitals according to a guideline
   - Is there a PBM clinical guideline?
     1. If yes, please describe
        - Full reference to PBM or not (eg. only anemia management etc.)

If not, do you know of any plans to introduce one

1. Do you consider PBM to be a ‘disruptive’ change for the healthcare system?
   *(‘disruptive’= change delivering a major improvement over the previous standard of care)*
2. Compared to other interventions how do you rank the importance/usefulness of PBM and why such rank? What do you consider more or equally important?
3. How good is the supply of blood components (red blood cells, fresh frozen plasma, platelets) in your country or organization? Where do you see the main issues currently with the supply of blood components?

| 1. Amount/scarcity |  |
| --- | --- |
| 1. Time to supply |  |
| 1. Quality /perceived safety |  |
| 1. Cost (e.g., the cost of PBM implementation is perceived to be higher; cost savings as a result of PBM implementation is not considered ....) |  |
| 1. Are there data available / published which would document the challenge(s)? Is the collection of these data mandatory? |  |
| 1. How is the supply of blood products in your country organized (e.g., centralized / decentralized) |  |
| 1. Are blood suppliers supportive of PBM or do they feel threatened? |  |
| 1. Who is paying for blood components (national level / hospital level) |  |
| 1. Who are the donors (e.g., Family replacement donation, other)? |  |
| 1. Any donor remuneration? |  |

**Section 2 (*PBM IS NOT YET INTRODUCED AS ROUTINE IN THE COUNTRY)***

1. How could PBM be introduced in the country or your organization?
   - What could be the main drivers for PBM – Why is PBM needed?
     *(Expected patient health outcomes; Issues which need to be addressed, e.g., issues with donated blood such as cost, supply, quality, transport, storage)?*
   - Who in your opinion will be the essential stakeholders who will have to be involved / convinced?
   - Which are in your experience or opinion the most important barriers which will have to be overcome? Please rate by marking the box (0 = not important, 4= highly important)

|  | **0** | **1** | **2** | **3** | **4** |
| --- | --- | --- | --- | --- | --- |
| Cost |  |  |  |  |  |
| Process / Feasibility |  |  |  |  |  |
| ‘Competition’ with other interventions |  |  |  |  |  |
| Collaboration / communication |  |  |  |  |  |
| Not sustainable |  |  |  |  |  |
| Too many stakeholders to manage |  |  |  |  |  |
| Change current work practice (too) difficult |  |  |  |  |  |
| Strong belief in transfusion practice |  |  |  |  |  |
| No or limited experience with PBM |  |  |  |  |  |

Please explain the 3 most important

1.

2.

3.

- - Are there data available in your country or organization which would help to support the argument that PBM policies or guidelines should be introduced?

|  | Published? Reference? | Source for data / |
| --- | --- | --- |
| cost / savings |  |  |
| Patient health outcome |  |  |
| quality or quality system |  |  |
| blood supply |  |  |
| …. |  |  |

- - In your opinion, which factors may be
    1. accelerating / supporting PBM implementation
    2. delaying / inhibiting PBM implementation

1. Would you think that structured case studies from other countries would help you to drive the implementation in your environment forward?
   - If yes: what would be important to show?
2. In your opinion, can patient organizations or advocates be part of PBM initiatives and if so, how?
   - Examples?
   - Are patient org’s in your country or organization aware of the difference? Do they have an experience they could share to develop ‘patient-based evidence’ or to establish patient preferences?
3. Do you have any additional remarks?

**SECTION 3 (*PBM IS IMPLEMENTED AS ROUTINE IN THE COUNTRY)***

1. When was PBM introduced in the country or your organization:
2. Are there any (local) data on the impact of introducing PBM in your country / organization which support the argument that PBM policies or guidelines should be introduced?

|  | Published? Reference? | Source for data / |
| --- | --- | --- |
| - cost / savings |  |  |
| - patient health outcome |  |  |
| - quality or quality system |  |  |
| - blood supply |  |  |
| - …. |  |  |

- - Would you like / are you planning to publish such evidence? (If so, please specify which evidence you are most interested in publishing)

1. How was PBM introduced in the country or your organization:
   - What were key learnings
   - What are/were the main drivers for PBM (Expected patient outcomes; Issues which had to be addressed, e.g., issues with donated blood such as cost, supply, quality, transport, storage)?
   - Who in your opinion were essential stakeholders who were involved?
   - Which are in your experience or opinion the most important barriers which had to be overcome? Please rate by marking the box (0 = not important, 4= highly important)

|  | **0** | **1** | **2** | **3** | **4** |
| --- | --- | --- | --- | --- | --- |
| Cost |  |  |  |  |  |
| Process / Feasibility |  |  |  |  |  |
| ‘Competition’ with other interventions |  |  |  |  |  |
| Collaboration / communication |  |  |  |  |  |
| Not sustainable |  |  |  |  |  |
| Too many stakeholders to manage |  |  |  |  |  |
| Change current work practice (too) difficult |  |  |  |  |  |
| Strong belief in transfusion practice |  |  |  |  |  |
| No or limited experience with PBM |  |  |  |  |  |

Please explain the 3 most important

1.

2.

3.

- - In your experience or opinion, which factors were or may be
    1. accelerating / supporting
    2. delaying / inhibiting
    3. what needs to be done to ensure sustainability

1. Based on your current knowledge, what would you recommend to others, who want to introduce PBM more broadly?
   - why
2. What is missing? Are there still current gaps and opportunities, which should be addressed?
   - Gaps
   - Opportunities
   - Cost and d. ability to embrace change
3. In your opinion, can patient organizations or advocates be part of PBM initiatives and if so, how?
   - Examples?
   - Are patients in your country or organization aware of the difference? Do they have an experience they could share to develop ‘patient-based evidence’ or to establish patient preferences?
4. Do you have any additional remarks or comments?
