# Supplementary material for: Making patient blood management the new norm(al) as experienced by implementors in diverse countries
Source: BMC Health Serv Res. 2021 Jul 2;21:634. doi: 10.1186/s12913-021-06484-3 (PMC8249439; doi:10.1186/s12913-021-06484-3)
Supplement: Supplementary file 3 — Additional file 2. [file 12913_2021_6484_MOESM2_ESM.pdf]

Domain 1: Research team and reflexivity Personal Characteristics

1. Interviewer/facilitator

Which author/s conducted the interview or focus group?

The majority of interviews were conducted by Anke-Peggy Holtorf. Two interviews with Chinese Implementors were translated and conducted by a graduate student in Chinese (Tianze Jiao; Pharmacy & Health economics, McGill University, Canada, with Chinese mother language). The second interviewer was intensively briefed by AP Holtorf. After the interviews, AP Holtorf was debriefed by T Jiao on the interview experience and output.

This is described in the methods section

2. Credentials

What were the researcher's credentials? E.g. PhD, MD

Dr. rer. nat, Dipl. Biol., MBA

3. Occupation

What was their occupation at the time of the study?

Managing Director and Analyst at Health Outcomes Strategies GmbH; Adjunct Faculty at the University of Utah, College of Pharmacy, Pharmacotherapy Outcomes Research Center

(indicated in the manuscript, title page)

4. Gender

Was the researcher male or female?

AP Holtorf is female (all except 2 interviews); T Jiao is male (2 interviews)

(manuscript, line 138-139)

5. Experience and training

What experience or training did the researcher have? Relationship with participants

Natural Sciences, Health economics & health policy, Previous research involving interviews.

The interviewer had no pre-existing relationship to the interviewees and was not known to any of the interviewees before.

N/A in manuscript,

6. Relationship established

Was a relationship established prior to study commencement?

The interviewees were first contacted by intermediates known to them (local experts from pharmaceutical industry who had participated in the pilot interviews, or Prof. A Hofmann) for their interest in participating in the interviews. If they agreed, they were contacted by the interviewer via an E-Mail, which explained the objectives and process of the interviews and contained an Internet-Link to register time slots if they were willed to participate. The interview questions were sent out to all registered participants at least a week before the interview by the interviewer.

7. Participant knowledge of the interviewer

What did the participants know about the researcher? e.g. personal goals, reasons for doing the research

The interviewer was introduced via E-Mail by name to the participants when the contact between the interviewer and the participants was established.
They received an explanation of the interview objectives and process with the first E-Mail from the interviewer. The interviewers introduced themselves at the beginning of the interview.
Where the interviews were conducted in Chinese language, both interviewers were introduced to the Chinese implementors and the implementors had the choice of who they preferred to talk to.
N/A in manuscript

8. Interviewer characteristics
What characteristics were reported about the interviewer/facilitator? e.g. Bias, assumptions, reasons, and interests in the research topic

The interviewer introduced their academic background and interests in the subject at the beginning of the interview.
Introduction in Additional File 1

Domain 2: study design. Theoretical framework

9. Methodological orientation and Theory
What methodological orientation was stated to underpin the study? e.g. grounded theory, discourse analysis, ethnography, phenomenology, content analysis

Content analysis (qualitative and quantitative). The interviewees were informed that the content of their responses would be evaluated for revealing potential commonalities and differences.
(They were also informed that it was planned to publish the findings in an international peer-reviewed journal and that their name and contribution through the interviews would be acknowledged in the publication and their consent was confirmed during the interview.

Participant selection

10. Sampling
How were participants selected? e.g. purposive, convenience, consecutive, snowball

The participants were selected for their leadership in implementing Patient Blood Management in their respective environments and their willingness to participate (purposive convenient sample). In a few cases (Mexico, Turkey), the first interviewee was asked for naming and making the link to additional implementors from that country (snowball).

11. Method of approach
How were participants approached? e.g. face-to-face, telephone, mail, email

The original contact was established via E-Mail.
The interviews were scheduled electronically (Doodle).
The interview was conducted by web-conference (GoToMeeting) or, if that was not possible for technical reasons, by telephone or WeChat (China): 29 interviews by web-conference, 5 by telephone, 2 by WeChat.

12. Sample size
How many participants were in the study?

11 pilot participants and 36 implementors from 12 countries (1-4 per country).

13. Non-participation

How many people refused to participate or dropped out? Reasons?

In addition to those implementors who were interviewed, 4 implementors from 4 countries never scheduled an interview date despite 2 reminders, and therefore, were not further contacted.

The reasons for not further responding are not known, but we assume that they were just too busy with their normal professional tasks or not interested due to other unknown reasons.

We only considered the implementors who scheduled an interview as ‘participants’. Of these, one interview could not be realized (drop-out) due to time-constraints of the implementor.

N/A in manuscript as not considered relevant to the research and results

Setting

14. Setting of data collection

Where was the data collected? e.g. home, clinic, workplace

It was left to the interviewee when and where the interview happened. Some did it from their private office during their off-work time, some from the workplace during their worktime.

N/A in manuscript as not considered relevant to the research and results

15. Presence of non-participants

Was anyone else present besides the participants and researchers?

Participation of other people was not excluded or encouraged before the interview. In 2 interviews (both in Mexico), another person (invited by the interviewee and sitting in the same room with the interviewee) was present to help the interviewees in case of language problems.

A few of the pilot interviews were conducted with a group of people (maximum 3) from pharmaceutical companies; usually with one main speaker.

N/A in manuscript as not considered relevant to the research and results

16. Description of sample

What are the important characteristics of the sample? e.g. demographic data, date

We were looking for **active implementors** in each of the target countries. All participants were known for their active engagement for Patient Blood Management in their respective environment. They could be clinical specialists, hospital managers, blood bank managers, or healthcare policy stakeholders.

Data collection

17. Interview guide

Were questions, prompts, guides provided by the authors? Was it pilot tested?

The interview was first designed by AP Holtorf and critically reviewed by A Hofmann. It was then pilot tested in 11 interviews with pharmaceutical industry experts (as listed in Table 1) and through an extensive interview with Prof DR Spahn, who had not been exposed to the interview before.

Only minor revision was deemed necessary after the piloting (e.g., there was 1 duplication in the barriers which was removed).
The pilot interviews served multiple purposes: 1.) to pilot the questionnaire, 2.) to train and standardize the interviewer for the interview flow, 3.) to lay the baseline knowledge for the country context for the interviewer.
(not included in the manuscript)

18. Repeat interviews
Were repeat interviews carried out? If yes, how many?

Each interview was individual and not repeated. However, the respondents received the interview notes and had the opportunity to add or change the contents as they deemed necessary. A few interviewees suggested minor corrections (language, clarification, or additional references).

19. Audio/visual recording
Did the research use audio or visual recording to collect the data?

Where the interviews could be conducted by web-conference (in 29 cases), the screen of the interviewer was shared, and the interviewees could see the notes that were taken. The discussions were recorded and the recordings were used to validate and correct the notes.

20. Field notes
Were field notes made during and/or after the interview or focus group?

The notes were taken during the interview. Directly after the interview (within a maximum of 76 hours), the notes were 'revised' (correcting typing mistakes, formulating full sentences, deleting duplications, etc.) with the help of the recordings.

21. Duration
What was the duration of the interviews or focus group?

Except 2 interviews, all other lasted between 45 and 60 minutes. One interview expanded to 120 minutes and the shortest interview took 35 minutes.

22. Data saturation
Was data saturation discussed?

N.A. as not considered relevant to the research and results

23. Transcripts returned
Were transcripts returned to participants for comment and/or correction?

Yes (see above)

Domain 3: analysis and findings

Data analysis

24. Number of data coders
How many data coders coded the data?

One (AP Holtorf evaluated and categorized all responses)

25. Description of the coding tree
Did authors provide a description of the coding tree?

The approach to evaluation is explained in the methods section as steps 1 to 4 (manuscript, Lines 146-151)
For the categories (coding), see results section (figures 1-3)

26. Derivation of themes
Were themes identified in advance or derived from the data?

Derived from data (manuscript, lines 146-152)

27. Software
What software, if applicable, was used to manage the data?

Numerical evaluation was done with an electronic spreadsheet.
(Manuscript, lines 150)

28. Participant checking
Did participants provide feedback on the findings?

Interviewees provided feedback on the notes relating to their own contribution.
The overall findings were not yet shared with the interviewees due to protect the ability to publish. They will be shared immediately after publication.
N/A in manuscript as not considered relevant to research and results

Reporting

29. Quotations presented
Were participant quotations presented to illustrate the themes / findings?
Was each quotation identified? e.g. participant number

There is only one direct quote in the manuscript, which is clearly marked as anonymous quote.

30. Data and findings consistent
Was there consistency between the data presented and the findings?

Yes, the data presented in Tables 1-4 and Figures 1-3 represent the data collected during the interviews. The categorization was derived from the responses.
Figure 4 presents and interprets the findings as a part of the discussion, which is also consistent with the results.

31. Clarity of major themes
Were major themes clearly presented in the findings?

Yes (see also frequency analysis in Figures 1-3)

32. Clarity of minor themes
Is there a description of diverse cases or discussion of minor themes?

Yes (see also frequency analysis in Figures 1-3) – however, a large congruency was found among all interviewees and there was no important diversion. Differences depended on country context, position of the implementor, and experience of the implementor. Not all implementors had experience in all levels of implementation.
